# Acute toxicity profile of craniospinal irradiation with intensity-modulated radiation therapy in children with medulloblastoma: A prospective analysis

**DOI:** 10.1186/s13014-015-0547-9

**Published:** 2015-11-24

**Authors:** Maurice C. Cox, Johannes M. Kusters, Corrie E. Gidding, Jolanda H. Schieving, Erik J. van Lindert, Johannes H. Kaanders, Geert O. Janssens

**Affiliations:** Department of Radiation Oncology, Radboud University Medical Centre, Nijmegen, The Netherlands; Department of Pediatric Oncology, Radboud University Medical Centre, Nijmegen, The Netherlands; Department of Neurology, Radboud University Medical Centre, Nijmegen, The Netherlands; Department of Neurosurgery, Radboud University Medical Centre, Nijmegen, The Netherlands; Department of Radiation Oncology, University Medical Center Utrecht and Princess Maxima Center for Pediatric Oncology, Utrecht, 3584 CX The Netherlands

**Keywords:** Craniospinal irradiation, Acute toxicity, IMRT, Medulloblastoma

## Abstract

**Background:**

To report on the acute toxicity in children with medulloblastoma undergoing intensity-modulated radiation therapy (IMRT) with daily intrafractionally modulated junctions.

**Methods:**

Newly diagnosed patients, aged 3–21, with standard-risk (SR) or high-risk (HR) medulloblastoma were eligible. A dose of 23.4 or 36.0Gy in daily fractions of 1.8Gy was prescribed to the craniospinal axis, followed by a boost to the primary tumor bed (54 or 55.8Gy) and metastases (39.6–55.8Gy), when indicated. Weekly, an intravenous bolus of vincristine was combined for patients with SR medulloblastoma and patients participating in the COG-ACNS-0332 study. Common toxicity criteria (CTC, version 2.0) focusing on skin, alopecia, voice changes, conjunctivitis, anorexia, dysphagia, gastro-intestinal symptoms, headache, fatigue and hematological changes were scored weekly during radiotherapy.

**Results:**

From 2010 to 2014, data from 15 consecutive patients (SR, *n* = 7; HR, *n* = 8) were collected. Within 72 h from onset of treatment, vomiting (66 %) and headache (46 %) occurred. During week 3 of treatment, a peak incidence in constipation (33 %) and abdominal pain/cramping (40 %) was observed, but only in the subgroup of patients (*n* = 9) receiving vincristine (constipation: 56 vs 0 %, *P* = .04; pain/cramping: 67 vs 0 %, *P* = .03). At week 6, 73 % of the patients developed faint erythema of the cranial skin with dry desquamation (40 %) or moist desquamation confined to the skin folds of the auricle (33 %). No reaction of the skin overlying the spinal target volume was observed.

**Conclusions:**

Headache at onset and gastro-intestinal toxicity, especially in patients receiving weekly vincristine, were the major complaints of patients with medulloblastoma undergoing craniospinal irradiation with IMRT.

## Background

The technique of craniospinal irradiation (CSI) is indicated for medulloblastoma/PNET-tumors and some more rare tumors with leptomeningeal spread like germ-cell tumors, atypical teratoid rhabdoid tumors and ependymoma [1–6]. The most common malignant embryonal tumor of the central nervous system in childhood is medulloblastoma [7, 8]. The treatment of medulloblastoma usually includes a combination of surgical resection, radiotherapy and chemotherapy [1, 2]. Currently, patients are categorized into standard-risk (SR) and high-risk (HR) groups. High-risk criteria include: positive cerebrospinal fluid cytology, or leptomeningeal metastasis on imaging, or residual tumor at primary site >1.5 cm^2^, or extra-axial metastases [9]. More recently, patients with large-cell anaplastic medulloblastoma are added [10]. Today, the technique most commonly used for treating the craniospinal axis (CSA) is a combination of two lateral opposed cranial fields, matched to a posterior field to treat the spine. This technique results in dose inhomogeneity, especially at the craniospinal junction and the spinal-spinal junction if required, depending on the thecal length [11]. The conventional technique also leads to a significant dose to structures anterior to the vertebra (thyroid, heart, lungs, bone marrow, intestine, kidneys) and the skin overlying the spine [11, 12].

In order to reduce the dose to organs at risk without decreasing the target coverage, other techniques for CSI are developed. CSI with electrons for the spinal part can be used as an alternative for photons in small children [13]. Both electron and proton beam radiation provide substantial sparing of non-target tissues anterior to the vertebra compared with conventional photon CSI [13–15]. Intensity Modulated Radiation Therapy (IMRT) results in better target coverage, improved junction homogeneity and a large gain in healthy tissue sparing [16–18]. In patients undergoing CSI with helical tomotherapy, junctions-related uncertainties even do not exist, because only one set-up point is needed during the whole treatment [19]. Highly-conformal photon techniques might result in a reduction of acute and late toxicity. Most data published on acute toxicity during CSI are retrospective and focus on a limited number of items [13, 19–22]. Prospective cumulative toxicity data on nausea, vomiting, headache, skin reactions and infections are available for a subgroup of patients with medulloblastoma treated with conventional CSI in the HIT-91 trial [23].

The purpose of this prospective study is to report on the acute toxicity of patients with medulloblastoma during CSI by IMRT.

## Methods

### Eligibility

Newly diagnosed patients, with medulloblastoma, aged 3 to 21, were eligible for the prospective registration of acute toxicity during CSI using IMRT with daily intrafractionally modulated junctions [18]. Toxicity items focusing on skin reactions, alopecia, voice changes, conjunctivitis, anorexia, dysphagia, vomiting, diarrhea, constipation, abdominal cramping, headache, fatigue and hematological changes were scored from baseline until week 10 after onset of radiotherapy, by an experienced pediatric radiation oncologist. The Common Toxicity Criteria used for this study (CTC, version 2.0) are provided in Table 1. Patients with SR or HR medulloblastoma, who received induction chemotherapy after surgery and before the onset of radiotherapy or patients receiving anesthesia during treatment, were excluded from the current analysis. Approval for the study was obtained from the Radboud University Medical Centre research Ethics Committee. Informed consent was obtained before data collection.

**Table 1:** National Cancer Institute Common Toxicity Criteria, version 2.0

Adverse event	Grade 0	Grade 1	Grade 2	Grade 3	Grade 4
Skin/radiation dermatitis	None	Faint erythema/dry desquamation	Moderate to brisk erythema or patchy desquamation mostly confined to skin folds	Confluent moist desquamation ≥1.5 cm diameter, not confined to skin folds, pitting edema	Skin necrosis, ulceration of full thickness, bleeding not induced by trauma or abrasion
Alopecia	Normal	Mild hair loss	Pronounced hair loss	-	-
Cough	Absent	Mild, relieve by non-prescription medication	Requiring narcotic antitussive	Severe cough or coughing spasms, poorly controlled or unresponsive to treatment	-
Voice changes (hoarseness, loss of voice)	Normal	Mild or intermittent hoarseness	Persistent hoarseness but able to vocalize, may have mild to moderate edema	Whispered speech, not able to vocalize, may have marked edema	Marked dyspnea/stridor requiring tracheostomy or intubation
Conjunctivitis	None	Ophthalmologic changes but asymptomatic (pain/irritation) or without visual impairment	Symptomatic/interfering with function but not interfering with activities of daily living	Symptomatic/interfering with activities of daily living	-
Anorexia	None	Loss of appetite	Oral intake significantly decreased	Requiring IV fluids	Requiring feeding tube or parenteral nutrition
Dysphagia	None	Mild, can eat regular diet	Requiring pureed, soft or liquid diet	Requiring feeding tube, IV hydration/alimentation	Complete obstruction, cannot swallow saliva
Vomiting	None	1x/24 h	2-5x/24 h	≥6x/24 h or need for IV fluids	Requiring parenteral nutrition, hemodynamic collapse, I.C.U.
Diarrhea	None	Increase ≤4x/24 h	4-6x/24 h or nocturnal stools	≥7x/24 h or incontinence, or need for parenteral support for dehydration	Hemodynamic collapse, I.C.U.
Constipation	None	Requiring stool softener or dietary modification	Requiring laxatives	Requiring manual evacuation or enema	Obstruction or megacolon
Abdominal pain or cramping	None	Mild; not interfering with function	Moderate; pain or analgesics, interfering with function but not with activities of daily living	Severe; pain or analgesics interfering with activities of daily living	-
Headache	None	Mild; non interfering with function	Moderate; pain or analgesics, interfering with function but not with activities of daily living	Severe; pain or analgesics interfering with activities of daily living	-
Fatigue/malaise	None	Increased fatigue, not altering normal activities	Moderate (decrease of performance status - 20 % in Lansky or Karnofsky); difficulty in performing some activities	Severe (decrease of performance status—40 % in Lansky or Karnofsky); loss of ability to perform some activities	Disabling

### IMRT technique

All patients underwent CSI with IMRT in supine position. Details of the procedure are described previously [18]. Briefly, treatment planning, using Pinnacle v.8.0 h software (Philips Medical Systems, Andover, MA), was based on a CT-scan obtained with a customized neck support (AccuForm Custom Cushions, Accuform, MED-TEC, Orange City, IA), a five-point fixation mask immobilization for the head (Efficast High- Precision Mask, Orfit Masks, Orfit Industries NV, Wijnegem, Belgium) and a cast for body fixation. The clinical target volume (CTV) was delineated comprising the entire brain and meninges for the cranial part. The spinal CTV contained the spinal canal as observed on CT-scan including the cerebrospinal fluid extension to the spinal ganglia. Based on MR-imaging, the inferior limit of the spinal CTV was defined at the caudal extent of the thecal sac. The spinal planning target volume (PTV) included an 8-mm margin in the caudal direction and a 5 mm margin in the lateral, anterior and posterior directions. For the cranial part of the PTV two parallel-opposed lateral photon fields were used, with segmental correction for overdose at the frontal and occipital area. Under- and overdosage of the craniospinal junction was prevented by including a daily intra-fractional beam displacement over a total length of 3 centimeters into the planning calculations. For this purpose, a 6-step junction was created by successively shifting the inferior borders of the two opposing cranial fields by 0.5 cm. This induced dose inhomogeneity was subsequently compensated by IMRT optimization of the adjacent spinal fields. The spinal IMRT beam arrangement consisted of five coplanar photon beams with gantry angles at 250°, 215°, 180°, 145°, and 110°. Subsequent to CSI, patients received a boost with IMRT or VMAT (Volumetric Modulated Arc Therapy) to the primary tumor bed. On indication, additional boosts to spinal and or intracranial metastases were delivered with IMRT.

### Radiation therapy

Radiotherapy was supposed to be initiated within 31 days following surgical resection. A dose of 23.4Gy or 36.0Gy in daily fractions of 1.8Gy was prescribed to the CSA for patients with SR- or HR medulloblastoma, respectively. Subsequently, a boost dose of 30.6Gy (total dose SR: 54.0Gy) or 19.8Gy (total dose HR: 55.8Gy) in daily fractions of 1.8Gy was planned to the primary tumor site. Concomitantly patients with HR disease did receive an additional boost to doses ranging from 39.6–55.8Gy to the spinal and/or intracranial metastases.

### Chemotherapy

Adjuvant chemotherapy, based on the regimen described by Packer et al., started 4 weeks after the end of radiotherapy [24]. A weekly intravenous bolus of vincristine (1.5–2.0 mg/m^2^) was combined with radiotherapy for patients with SR medulloblastoma and HR medulloblastoma participating in the COG-ACNS-0332 trial [10].

### Statistics

Statistical analyses were performed with SPSS 21.0. Descriptive statistics were used to calculate median values (and 95 % confidence intervals) of patient, tumor and treatment characteristics. The Chi-square test and Fisher’s exact test were used to compare toxicity outcomes between different groups.

## Results and discussion

### Patient and treatment characteristics

Between March 2010 and December 2014, data from 19 consecutive patients with newly diagnosed medulloblastoma at the Radboud University Medical Centre, were collected. Four patients were excluded from analysis for reasons of induction chemotherapy (*n* = 3) and anesthesia (*n* = 1). The baseline patient and treatment characteristics are listed in Table 2.

**Table 2:** Patient and treatment characteristics

Patient and treatment characteristics	
Sex (n)	
Male	8
Female	7
Age at diagnosis (years)	
Median	8
Range	4–16
Staging (n)	
SR	7
HR M0	0
HR M1	4
HR M2	1
HR M3	3
Time between surgery and start RT (days)	
Median	31
Range	15–43
95 % CI median	29–32
CSI dose (n)	
23.4 Gy/1.8	7
36.0 Gy/1.8	8
Patients receiving cranial/spinal boost (n)	4
Cranial boost	3
Spinal boost	3
Overall treatment time RT (days)	
Median	42
Range	39–43
95 % CI median	41–42
Patients with treatment interruptions (n)	1
Treatment interruptions (days)	1
Chemotherapy (n)	
SR (Concomitant and Adjuvant)	7
HR (Concomitant and Adjuvant)	2
HR (Adjuvant only)	6

The median age of the patient group was 8 years (range, 4–16 years). Radiation therapy started within 32 days from surgery in 14 of 15 children (median, 31 days; 95%CI: 29–32 days; range, 15–42 days). All children completed radiotherapy within 43 days (median, 42 days; 95%CI: 41–42 days; range, 39–43 days). Radiation therapy was interrupted for 1 day in one patient, due to lack of compliance. A weekly bolus of vincristine was given to all children with SR disease (*n* = 7). Two out of eight children with HR disease, participating in the COG-ACNS-0332 trial, received vincristine. Four patients with HR disease did receive additional boost doses to spinal and or intracranial metastases.

### Overall toxicity profile

**Fig. 1:**
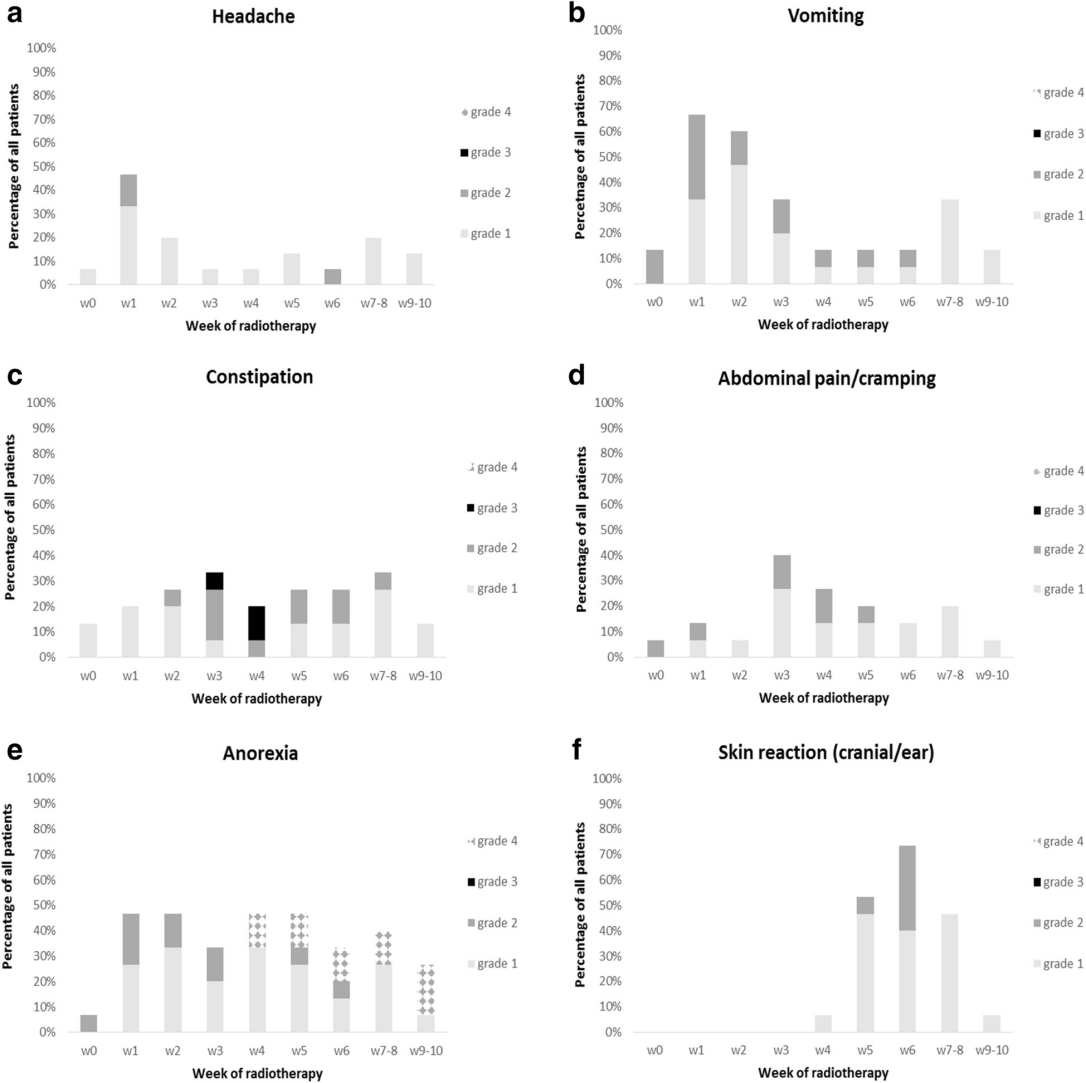
Toxicity, scored from baseline (w0) until week 10 after onset of radiotherapy, in patients with standard-risk and high-risk medulloblastoma

Within 72 h from start of treatment, headache and vomiting were observed in 46 and 66 % of children, respectively (Fig. 1a and b). A peak incidence in constipation (33 %) was observed during week 3 (Fig. 1c). From the third week of radiotherapy, 40 % of the children experienced mild (grade 1) or moderate (grade 2) abdominal pain/cramping (Fig. 1d). Anorexia was observed during the whole period of radiation treatment (Fig. 1e).

Pronounced hair loss occurred in all 15 patients from week 3. At week 6, 73 % of the patients developed faint erythema of the occipital part of the cranial skin combined with dry desquamation (40 %), or moist desquamation confined to the skin folds of the auricle (33 %) (Fig. 1f). Radiation dermatitis of the skin overlying the spinal target volume was not observed. Conjunctivitis was not observed and dysphagia (grade 1), cough (grade 1) and voice changes (grade 1) were limited to 13, 20 and 13 % of all patients, respectively. Severe diarrhea (grade 3) was recorded in 1 patient, as a result of a Salmonella infection.

Before start of radiation therapy, 13 % of the patients received treatment with corticosteroids. To relieve side effects of treatment, dexamethasone and ondansetron were prescribed. From week one to six (w1-6) low doses of dexamethason (range, 0.5–3.0 mg per day) were used in 60, 47, 53, 33, 40 and 40 % of patients, respectively. Ondansetron was used by 47 % (w1), 53 % (w2), 53 % (w3), 47 % (w4), 40 % (w5) and 33 % (w6) of patients.

Without any use of hematopoiesis stimulating factors, the median value of white blood cells and platelet count at nadir was 2,2 · 10^9^/L (range: 1,2–3,1; normal: 4,0–10) and 121 · 10^9^/L (range: 41–177; normal: 150–400), respectively.

### Toxicity profile in relation to medulloblastoma risk groups

**Fig. 2:**
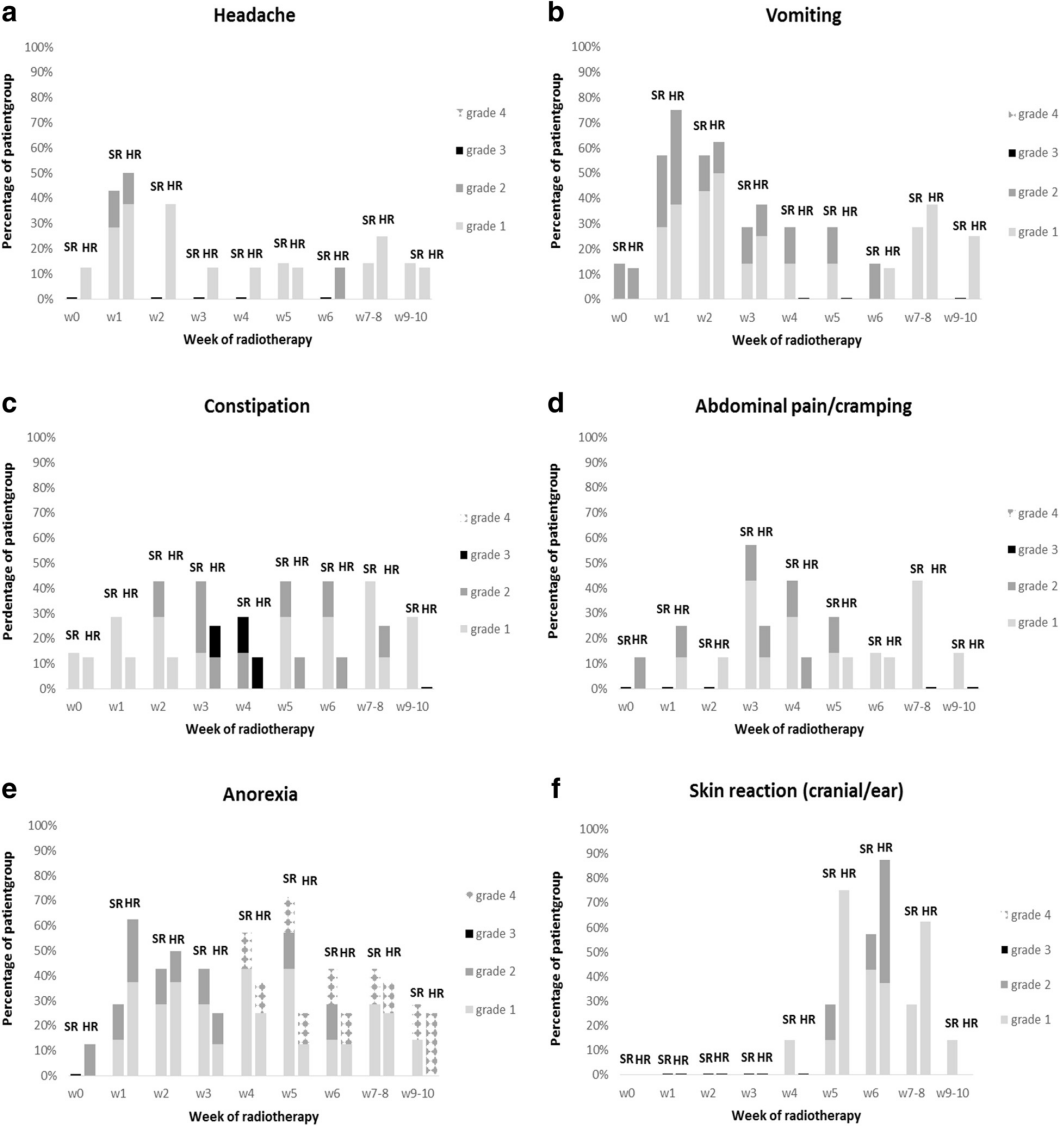
Toxicity, scored from baseline (w0) until week 10 after onset of radiotherapy, in patients with standard-risk (SR) versus high-risk (HR) medulloblastoma. No significant differences were observed between the two groups

The acute toxicity profile for headache, skin toxicity, anorexia, vomiting, constipation and abdominal cramping in patients with SR vs HR medulloblastoma is illustrated in Fig. 2. Although none of the toxicity items scored were significantly different between the SR and HR group, there was a trend (*P* = 0.13) towards an increased incidence of cranial/ear skin toxicity during the fifth week in the HR group.

### Toxicity profile in relation to intravenous vincristine use

**Fig. 3:**
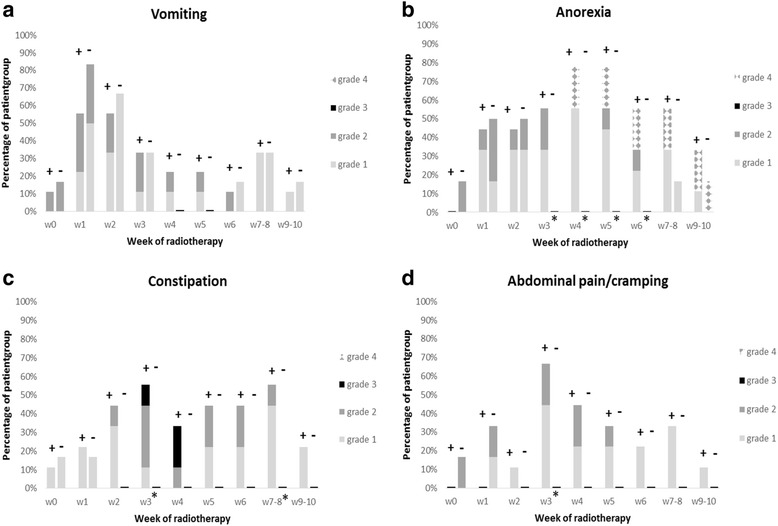
Toxicity, scored from baseline (w0) until week 10 after onset of radiotherapy, in patients well (+) or not (−) receiving concomitant intravenous vincristine. Statistically significant changes (*p* < 0.05) are indicated by *

A comparison of acute gastro-intestinal toxicity (anorexia, abdominal pain/cramping, constipation and vomiting) in patients with (*n* = 9) and without (*n* = 6) concomitant intravenous vincristine is shown in Fig. 3. Anorexia was observed more frequently in patients receiving vincristine during weeks 3 to 6. During week 3, constipation and abdominal pain/cramping occurred more frequently in the vincristine group (Fig. 3c and d). No significant difference in incidence of vomiting, skin reaction or headache was observed between the groups with and without vincristine.

## Discussion

Gastro-intestinal toxicity was the major complaint during radiotherapy in children with medulloblastoma undergoing CSI with IMRT. Subgroup analysis revealed the use of intravenous vincristine as main reason for gastro-intestinal toxicity. Headache and vomiting were observed at the onset of treatment.

Within 72 h from the start of treatment, headache and vomiting were reported in a large number of patients. In a retrospective study of Suneja et al. headache was observed during radiation treatment in 50 % of children with mainly medulloblastoma treated by proton beam therapy to the CSA [22]. Incidence peaked during the first 2 weeks of the treatment course [22]. The incidence of nausea and vomiting was 50 and 25 %, respectively [22]. In a group of 14 patients with medulloblastoma undergoing CSI by 3D conformal- and tomotherapy in combination with vincristine (*N* = 12/14), Huang et al. observed nausea/vomiting during the course of treatment in 64 % of patients [21]. The combination of headache and vomiting at the onset of radiotherapy in patients treated with photons and protons, does suggest that cerebral edema resulting in increased intracranial pressure, was the main reason for these symptoms [25]. In order to reduce headache and vomiting, patients are given paracetamol, anti-emetics, dexamethasone or a combination of both [26]. Before start of radiation therapy, 13 % of patients were dependent of steroids. During the first week of radiotherapy, 60 % of the children needed dexamethasone to relieve symptoms. According to a double-blinded placebo-controlled randomized trial by Wong et al., the addition of dexamethasone to ondansetron as prophylaxis for radiation induced emesis from radiotherapy to the upper abdomen, resulted into a significant improvement of complete control of emesis during the first 2 weeks of fractionated radiotherapy [27].

Constipation and abdominal pain were only observed in patients receiving vincristine combined with radiotherapy and mostly from the third week of treatment. Anorexia was observed during the whole period of radiation treatment. However, from week 3 it was observed only in the group of patients receiving combined treatment. Suneja et al. demonstrated anorexia during the course of treatment in 83 % of patients treated with proton beam therapy to the CSA [22]. Although significant higher radiotherapy doses were delivered to the CSA in patients with HR compared to SR medulloblastoma, there were no significant differences observed in anorexia, constipation and abdominal cramping. In contrast, almost 80 % (versus 0 %) of patients receiving vincristine developed a combination of these symptoms. Acute gastro-intestinal toxicity during radiotherapy was mainly related to the concomitant use of vincristine and independent of the radiotherapy dose. Neurotoxicity of the gastro-intestinal tract, resulting in constipation and/or abdominal pain, is a well-known side-effect of vincristine [28]. These symptoms are most prominent approximately 3 to 10 days after drug administration and usually resolve within several days after discontinuation of chemotherapy. The impaired motility of the intestines is dose related and most prominent if doses larger than 2.0 mg/m^2^ per bolus are used [28]. In the current analysis, vincristine related side-effects as constipation and abdominal pain were mainly observed from the third week of radiation treatment. Optimization of supportive care with laxatives can probably explain the decrease in abdominal pain/cramping from week 4. Neurotoxicity can be enhanced by the concomitant use of anti-emetics, such as ondansetron and granisetron [29]. From the second week of radiotherapy, 54 % of the children received one or a combination of these medications to relieve gastro-intestinal symptoms. Randomized controlled trials concerning prevention and/or management of chemotherapy-induced constipation in oncology patients are absent.

In line with Suneja et al. alopecia occurred in all patients from week 3 of treatment [22]. At week 6, 73 % of patients developed faint erythema of the occipital area of the cranial skin. In some of them, erythema was combined with dry desquamation (40 %), or patchy desquamation confined to the skin folds of the auricle (33 %). A trend towards more skin toxicity was observed in patients with HR medulloblastoma. In contrast, radiation dermatitis of the skin overlying the spinal target volume was not observed. Kortmann et al. reported a comparable incidence of mild or marked erythema (±75 %) in children with SR and HR medulloblastoma treated with a conventional CSI technique, without any significant difference between patients receiving neoadjuvant or concomitant chemotherapy [23]. According to Suneja et al., 100 % of the patients receiving proton CSI developed grade 1 or 2 dermatitis (CTC, version 2.0) [22]. A benefit in radiation dermatitis in favor of photons compared to protons can be expected. An explanation for the lack of radiation dermatitis overlying the spinal skin observed in patients from the current analysis can be explained by the 5-beam arrangement with photons significantly reducing the dose close to the skin [18]. For the cranial part, with a target volume close to the skin, also a higher entrance dose is expected from proton beams compared to photon beams [14, 30]. Hair loss may be permanent with total doses greater than 40 Gy [31].

Overall treatment time is a prognostic factor for progression-free survival in patients with medulloblastoma [32, 33]. All our patients completed radiotherapy within 43 days. Due to poor compliance one patient resumed treatment after 1 day. Chang et al. reported interruptions of more than 3 days in 5 % of children treated with electrons or photons [13]. In the multicenter HIT-91 trial, Kortmann et al. observed interruptions of radiotherapy in 33 % of patients receiving induction chemotherapy compared to 19 % of patients in the maintenance chemotherapy arm [23]. This corresponded to a mean protraction of overall treatment time of 11.5 and 7.5 days, respectively. The main reasons for a different number of treatment interruptions observed in literature may be multifactorial: myelosuppression due to chemotherapy before radiotherapy, bone marrow sparing radiotherapy techniques, multicenter versus single center studies, the use of different hematological criteria for treatment interruption, and the quality of supportive care.

Although not an objective of this study, late toxicity remains a major issue after treatment for medulloblastoma. Secondary malignancy is one of the endpoints that needs attention when using highly-conformal photon techniques with larger areas of low-dose irradiation compared to the conventional techniques. Recently, the 10-year follow-up data on second malignancy after a combination of conventional radiotherapy and chemotherapy for 379 patients with non-disseminated medulloblastoma were published [34]. Interestingly, the majority of these second malignancies (at least 11 of 15) developed within the target volume of the craniospinal axis (*N* = 7), hematopoietic tissues (*N* = 3) or bone (*N* = 1) and will not be altered by treatment technique.

## Conclusion

Headache and vomiting at onset, and gastro-intestinal toxicity during treatment were the major complaints in children with medulloblastoma undergoing CSI with IMRT. Subgroup analysis revealed the use of intravenous vincristine as main reason for gastro-intestinal toxicity. In an attempt to compare data from literature, the majority of items scored during radiotherapy seem to be independent of treatment technique used. Compared to protons, electrons and conventional techniques with photons, a benefit in favor of IMRT is observed for skin toxicity, especially for the spinal part.

## Abbreviations

IMRT: Intensity-modulated radiation therapy
SR: Standard-risk
HR: High risk
CSI: Craniospinal irradiation
CSA: Craniospinal axis
CTV: Clinical target volume
PTV: Planning target volume
VMAT: Volumetric modulated arc therapy
